# Photogenerated-hole-induced rapid elimination of solid tumors by the supramolecular porphyrin photocatalyst

**DOI:** 10.1093/nsr/nwaa155

**Published:** 2020-07-02

**Authors:** Zijian Zhang, Li Wang, Weixu Liu, Zihe Yan, Yongfa Zhu, Shuyun Zhou, Shanyue Guan

**Affiliations:** Department of Chemistry, Tsinghua University, Beijing 100084, China; Key Laboratory of Photochemical Conversion and Optoelectronic Materials, Technical Institute of Physics and Chemistry, Chinese Academy of Sciences, Beijing 100190, China; Department of Chemistry, Tsinghua University, Beijing 100084, China; Department of Chemistry, Tsinghua University, Beijing 100084, China; Department of Chemistry, Tsinghua University, Beijing 100084, China; Key Laboratory of Photochemical Conversion and Optoelectronic Materials, Technical Institute of Physics and Chemistry, Chinese Academy of Sciences, Beijing 100190, China; Key Laboratory of Photochemical Conversion and Optoelectronic Materials, Technical Institute of Physics and Chemistry, Chinese Academy of Sciences, Beijing 100190, China

**Keywords:** supramolecular photocatalyst, photogenerated holes, solid tumor, rapid elimination, red light

## Abstract

The rapid, complete, targeted and safe treatment for tumors remains a key issue in cancer therapy. A novel treatment of solid tumors by supramolecular photocatalyst Nano-SA-TCPP with the irradiation of 600–700 nm wavelength is established. Solid tumors (100 mm^3^) can be eliminated within 10 min. The 50-day mouse survival rate was increased from 0% to 100% after the photocatalytic therapy. The breakthrough was owing to the cell membrane rupture and the cytoplasmic loss caused by photogenerated holes inside cancer cells. The porphyrin-based photocatalysts can be internalized in a targeted manner by cancer cells due to the size selection effect, without entering the normal cells. The therapy has no toxicity or side effects for normal cells and organisms. Moreover, the photocatalytic therapy is effective for a variety of cancer cell lines. Because of its high efficiency, safety and universality, the photocatalytic therapy provides us with a new lancet to conquer the tumor.

## Teaser

The solid tumor (about 100 mm^3^) can be eliminated rapidly via the novel photocatalytic therapy induced by photogenerated holes of the supramolecular porphyrin photocatalyst.

## INTRODUCTION

According to the latest statistics, cancer is still a heavy threat to public health worldwide [1]. There is still an urgent need for new approaches to cancer treatment [2]. Currently, it is still difficult to cure cancers with most clinical cancer therapies, which also have serious side effects [3–5] and result in opportunities for metastasis, spread and drug resistance mutations. Nanomedicine for cancer therapy has been extensively developed for preclinical and clinical applications, especially with phototherapeutic approaches which could be precisely targeted at tumors and minimize damage to normal cells [6–9].

Photothermal therapy (PTT) utilized the photothermal effect of nanoparticles to produce local heat above 42°C to kill cancer cells [10]. PTT has some intriguing and unique advantages such as its minor invasiveness and high effectiveness [11]. However, the metallic and carbon-based nanomaterials of photothermal agents (PTAs) are difficult to metabolize, causing cumulative toxicity and permanent damage to the brain, kidney, liver and other organs [12,13]. And the organic small molecules of PTAs, and the relatively low photothermal stability and photothermal conversion efficiency (PCE) restrict its clinical application [14]. As for other PTAs like inorganic 2D materials and polymer nanoparticles, their synthetic strategy is too complicated and usually has wide size distribution [15].

The other clinically approved phototherapy is photodynamic therapy (PDT), employing exogenous reactive oxygen species (ROS) generated from light activating photosensitizers (PSs) and oxygen to kill cancer cells [16,17]. Because ROSs are chemically reactive radicals or non-radical molecules derived from oxygen molecules [18], PDT is an oxygen-dependent process and can damage a wide range of cancer cells. As a result, there is no drug resistance for repeated PDT treatments [19]. In spite of these advantages, it is often inefficient and requires repeated treatments, due to the need of oxidative intermediate species in the hypoxic tumor microenvironment [20,21].

Photocatalysis, driven by the energy of photons, oxidizes or reduces substrate molecules. Recently, photocatalysts have been applied in life-related antibacterial [22,23] and antiviral [24] fields, which inspired us to suppose that photocatalysts might have functions in tumor therapy. Not long ago, we reported a method for preparing supramolecular photocatalysts of self-assembled tetra-carboxyphenyl porphyrin (SA-TCPP) and demonstrated the amazing oxidizing ability of its photogenerated holes excited by light of 420–750 nm wavelength [25]. And porphyrin-based molecular drugs have been widely used in PDT [26] owing to porphyrin's excellent biocapacity and singlet oxygen evolution; some of these drugs have already achieved clinical application. It is well known that one of the barriers of the phototherapeutic approach is its penetration depth, which is also significant for detection. And the red/near infrared (NIR) light region between 600 and 1200 nm is called the optical window of tissue [27–29], which is beneficial for deep penetration. Considering the above, we tried to utilize the photogenerated holes of the SA-TCPP to achieve a strong oxidative killing of solid tumors and form a theragnostic system for cancer cells. If the idea is feasible, it could be more efficient than PDT and PTT because it does not need heat and oxygen, which is more suitable for the tumor microenvironment.

## RESULTS AND DISCUSSION

### The characterization of the Nano-SA-TCPP

**Figure 1.**
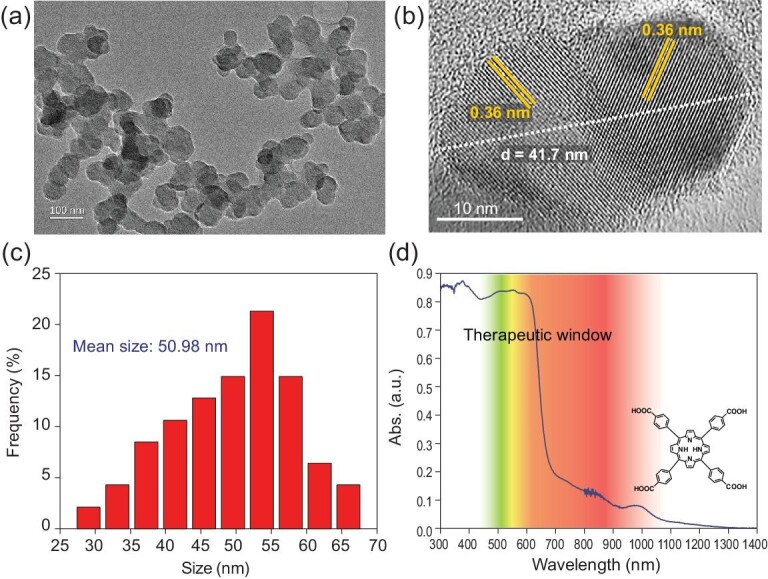
The characterization of the Nano-SA-TCPP. (a) The morphology of the Nano-SA-TCPP observed by TEM; (b) The HRTEM image of Nano-SA-TCPP; (c) The particle size distribution of the Nano-SA-TCPP; (d) The UV-Vis-NIR absorption spectrum of the Nano-SA-TCPP and the molecular structure of TCPP.

The supramolecular Nano-SA-TCPP was prepared as previously reported [25], and carefully characterized. The specific methods are emphasized in the Supplementary Data. Generally, the SA-TCPP photocatalyst is a crystalline nanoplate as shown in Fig. 1a, thus it is named Nano-SA-TCPP in the article. The excellent crystallization properties of the supramolecular material can be observed with high-resolution transmitting electron microscopy (HRTEM) as shown in Fig. 1b, where the clear d-spacing of 0.36 nm proved the π-π stacking interaction between TCPP molecules. And the particle size distribution was further counted as shown in Fig. 1c, which calculated that the mean particle size of the material is about 50 nm. Furthermore, the widened peaks in the X-ray diffraction spectrum (Fig. S1) also indicated the nano size of the material particles and the π-π interaction. The material was also analyzed with the IR spectrum (Fig. S2) to confirm the correct preparation. Owing to the fantastic molecular structures of carboxyl groups and conjugated central ring in TCPP, the Nano-SA-TCPP presents negative Zeta potential of −90.3 mV (Fig. S3) and amphiphilic properties (Fig. S4), which can be dispersed homogeneously in water for cell culture. Thus, the properties above mentioned, the nano size, negative surface potential [30] and amphiphilic ability [31] contributed to the phagocytosis of the supramolecular Nano-SA-TCPP photocatalyst by cancer cells. Moreover, benefiting from the large internal delocalization system of the TCPP molecule, the supramolecular Nano-SA-TCPP presents a wide absorption spectrum, as shown in Fig. 1d. It can be observed that the absorption range reached NIR. The longer wavelength is beneficial for penetrating biological tissue into the targeted region. And the region of 600–1200 nm is considered as a therapeutic window because of the lesser absorption by biological tissues [32]. Thus, the Nano-SA-TCPP *in vivo* can be induced by red light to conduct a powerful anti-cancer performance.

### The rapid and complete solid tumor elimination with Nano-SA-TCPP under red light

**Figure 2.**
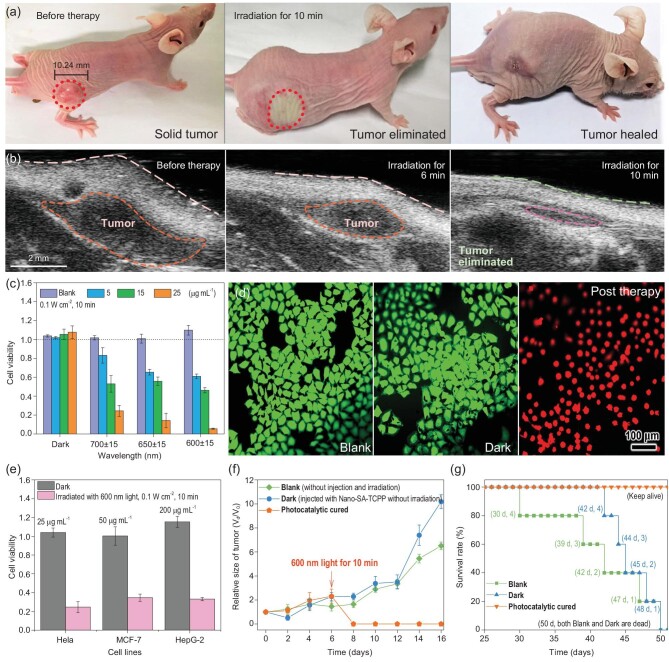
The photocatalytic cancer therapy with Nano-SA-TCPP. (a) The photographs of photocatalytic therapy for tumor-bearing mice (irradiation wavelength and power: 600 nm, 0.1 W cm^−2^). (b) The ultrasound image of tumors before and post photocatalytic cancer therapy (irradiation wavelength and power: 600 nm, 0.1 W cm^−2^). (c) Hela cells viability with different concentrations of Nano-SA-TCPP under irradiation of 0.1 W cm^−2^ for 10 min. (d) The green fluorescence image of living Hela cells (Blank and Dark) and killed cells (post therapy, irradiation wavelength and power: 600 nm, 0.1 W cm^−2^). (e) Photocatalytic cancer therapy with Nano-SA-TCPP dispersions to three types of different cancer cell lines (irradiation wavelength and power: 600 nm, 0.1 W cm^−2^). (f) Relative tumor sizes of the experimental mice. (g) The statistical analysis of the experimental mouse survival rate.

On this basis, we tested the solid tumor eliminating performance *in vivo* with the supramolecular photocatalyst, as shown in Fig. 2a and b, and Movie S1. The specific methods are introduced in Methods and the Supplementary Data. The light over 600 nm is known to be able to penetrate the skin over a range of 10 mm [27,33]. As characterized, the Nano-SA-TCPP can absorb the light up to 1200 nm. Therefore, we used a subcutaneous tumor model about 100 mm^3^ to test the treatment effect. The tumor site was irradiated with a 600 nm light of 0.1 W cm^−2^ for 10 minutes after injection of Nano-SA-TCPP. The treatment process is shown in Figs S5 and S6. The solid tumor site was atrophied and flattened obviously, where the original convex site had disappeared. On the second day, the irradiated part was scarred, and fell off one week later, revealing the newly reconvened tissue. We also irradiated the healthy mouse with the same condition as shown in Fig. S7, which did not present obvious change. The results also confirm the safety of the light source and indicated the cancer therapy effect of the Nano-SA-TCPP.

To verify the anticancer performance under the red light, control experiments *in vitro* have also been investigated. Hela cells were incubated with dispersions of Nano-SA-TCPP and irradiated under 600–700 nm. It can be seen that the Nano-SA-TCPP can significantly inhibit the proliferation of Hela cells under the red light irradiation (0.1 W cm^−2^), as shown in Fig. 2c. Nano-SA-TCPP dispersion of 25 μg mL^−1^ can completely kill the Hela cells within 10 minutes under 600 nm. It should be pointed out that the killing performance was not solely caused by the light irradiation, where the irradiated cells without the Nano-SA-TCPP (marked by ‘Blank’ in the following) did not present decreased viability. To visualize the therapy, Hela cells were stained by applying calcein-AM and propidium iodide (Calcein-AM/PI), as shown in Fig. 2d. The strong green fluorescence of living Blank Hela cells can be observed. Similarly, the Hela cells incubated by Nano-SA-TCPP without irradiation (marked by ‘Dark’ in the following) can stay alive and look similar to the Blank group. After irradiation with 600 nm light for 10 min, the Hela cells were killed and presented a strong red fluorescence and the spherical morphology. Moreover, the number of intact cells in the visual field was significantly reduced, which means that the cells may have ruptured during the photocatalytic therapy. The results confirm that the light acts like a switch with regard to anticancer performance; only under irradiation can the photocatalyst conduct the strong oxidative anticancer effect. Moreover, the therapy is not only for Hela cells but also had significant effects on other cancer cell lines such as MCF-7 and HepG-2, as shown in Fig. 2e. Because of the different cellular properties, these cell lines presented obvious differences in tolerance.

Long-term therapy has been investigated as well. Relative tumor volumes were calculated from the tracked volumes (Fig. S8) to further validate the therapeutic effects and tumor proliferation, as shown in Fig. 2f. For the photocatalytic cured group, there was no solid tumor observed anymore, proving the complete therapy. While, in the Dark group, the solid tumor grew wildly, same as the Blank group. And the anatomy and sectioning results (Figs S9 and S10) can more accurately confirm the complete therapeutic effect on solid tumors. Obvious subcutaneous tumors were found in the control groups, while only scars and muscles were observed in the cured group with Nano-SA-TCPP. Thus, the 50-day survival rate of the photocatalytic cured mice was increased compared to the control groups, as shown in Fig. 2g. On the 50th day of the experiment, the treated mice remained healthy, while the untreated control group had all died.

### The targeted property of the Nano-SA-TCPP

**Figure 3.**
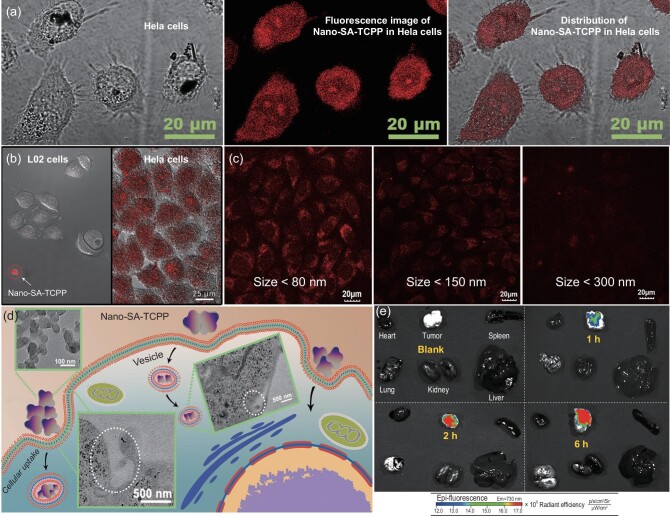
The cellular uptake and targeted property of Nano-SA-TCPP. (a) The Nano-SA-TCPP distribution in Hela cells observed by fluorescence image. (b) The comparison of cellular uptake behavior between normal cells (L02) and Hela cells by the fluorescence image of Nano-SA-TCPP. (c) The cellular uptake behavior of Nano-SA-TCPP with different particle sizes. (d) The cellular uptake process of Nano-SA-TCPP, where the inserted images are the TEM images of Nano-SA-TCPP and cells in the uptake process. (e) The Nano-SA-TCPP distribution in major organs and the tumor.

The targeted property of the supramolecular photocatalyst has been further explored as well. The Nano-SA-TCPP can get into the cancer cells and be distributed in the cytoplasm evenly as shown in Figs 3a, S11 and S12. Meanwhile, the concentrations of Nano-SA-TCPP in Hela cells and normal L02 cells presented an obvious difference, as shown in Fig. 3b. The Hela cells presented strong fluorescence indicating the large-amount internalization of Nano-SA-TCPP. While in the L02 cells, the Nano-SA-TCPP was blocked outside the cell. The same result has also been observed and proved by Peng *etal.* [34]. It is reported that the targeting property is caused by the size selection effect of cellular phagocytosis between cancer cells and normal cells [35,36].

Indeed, the cellular uptake amount significantly depended on the nanoparticle size, as shown in Fig. 3c. Different centrifugal speeds were applied to classify the particle size of the Nano-SA-TCPP material, where the smaller particles enter the cells more efficiently. Besides, the internalization process can be observed as shown in Figs 3d and S13–S16. The Nano-SA-TCPP can be internalized and transported by vesicles. This also makes the nanomaterials used in photocatalytic therapy show the cumulative targeting of cancer cells [37]. The targeting property *in vivo* was further tested by injection of Nano-SA-TCPP dispersion (Fig. S17) through the tail vein. As shown in Fig. 3e, the Nano-SA-TCPP can be observed enriched in the solid tumor site due to its targeting and enhanced permeability and retention effect [38]. The fluorescence intensity of the tumor site was significantly higher than that of other organs, indicating that the material targeted the tumor. The targeting property provides a safety guarantee to healthy cells and organs.

### The biocompatibility and biosafety of Nano-SA-TCPP

**Figure 4.**
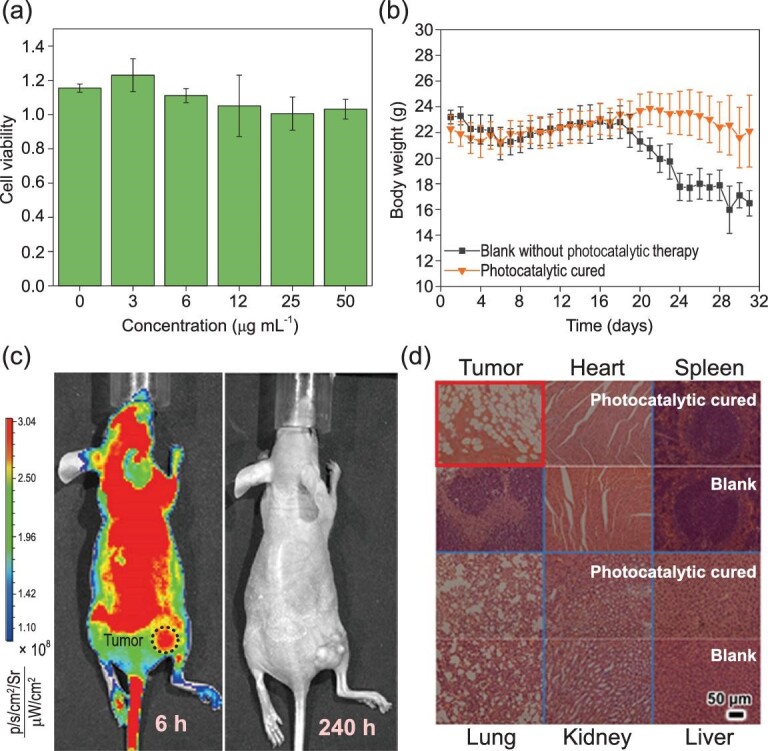
The biosafety of the Nano-SA-TCPP. (a) Cell viability of Hela cells with different concentrations of Nano-SA-TCPP. (b) The statistical body weight analysis of the experimental mice. (c) The Nano-SA-TCPP distribution and metabolization in the whole body observed by the fluorescence image of Nano-SA-TCPP. (d) Histology analysis of the major organs and tumors after treatment.

The biocompatibility of the photocatalytic therapy was also revealed. The Nano-SA-TCPP dispersions of different concentrations were incubated with Hela cells. The concentration of Nano-SA-TCPP below 50 μg mL^−1^ has no significant side effect on the proliferation, as shown in Fig. 4a, which is the basis for clinical application. And in the experimental term, the live quality of mice was not affected as shown in Fig. 4b. The body weight of the photocatalytic cured group was significantly increased, whereas in the other untreated control groups it dropped obviously. And the untreated groups became noticeably thin.

Due to supramolecular Nano-SA-TCPP having no metallic elements, the Nano-SA-TCPP can be completely metabolized, as shown in Figs 4c and S18–S20. The amount of Nano-SA-TCPP in the tumor site was first increased and then decreased, indicating that the organic materials did not cause cumulative toxicity. The maximum concentration in the tumor site appeared at 6 hours after injection. And the maximum concentration in the whole body appeared at 24 hours after the injection of the drug, which was caused by the lag of systemic metabolism. Then, as the observation time was prolonged, 240 hours later the Nano-SA-TCPP was completely metabolized and the fluorescence recovered to the background level in the whole body.

As shown in Fig. 4d, the injection of Nano-SA-TCPP did no harm to the main organs. The photocatalytic cured mice stayed healthy and the solid tumor was eliminated. In the controlled group, the solid tumor grew crazily. The results of such experiments demonstrate the striking therapeutic effect on solid tumors by photocatalytic therapy, eliminating the solid tumor tissue within 10 minutes. Such excellent therapeutic effects and biosafety are not available in any conventional phototherapy method.

### The mechanism of photogenerated-hole-induced cancer therapy

**Figure 5.**
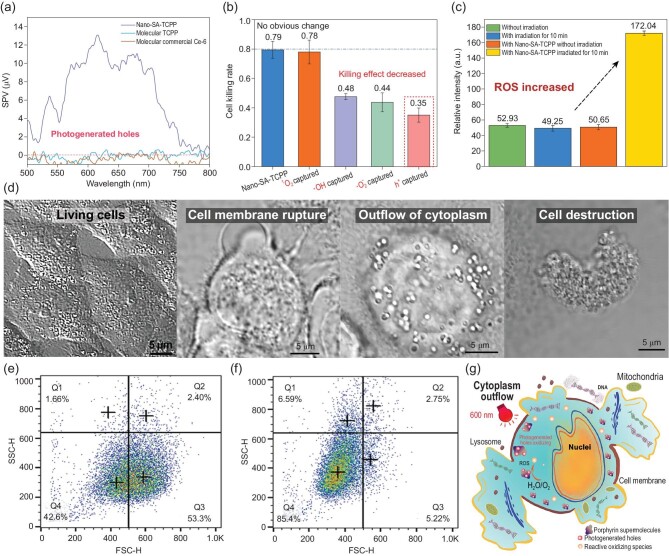
The mechanism of photocatalytic cancer therapy by Nano-SA-TCPP. (a) The detection of photogenerated holes on the surface of the Nano-SA-TCPP outside of the cancer cells. (b) The reactive species capture in cancer cells. (c) The concentration changes of ROS inner cells with the probe of DCFH-DA. All the experiments were repeated three times and calculated the mean values and error bar. (d) The photographs of cancer cells before and after photocatalytic therapy. (e) Flow cytometry analysis of Hela cells incubated by Nano-SA-TCPP without irradiation. (f) Flow cytometry analysis of Hela cells incubated by Nano-SA-TCPP with irradiation for 10 min. (g) The mechanism simulation diagram of photocatalytic therapy.

With the huge breakthrough, the mechanism was deeply revealed. The outstanding performance is from the huge innovation in the therapy mechanism of photogenerated hole oxidation. Photogenerated holes are high-energy oxidation states formed on the semiconductor photocatalyst surface after excitation by light [39]. The oxidizing ability of photogenerated holes is so strong that it can destroy small molecules [40]. The photocatalytic therapy utilizes highly oxidative photogenerated holes, which is different from the traditional PDT based on the singlet oxygen. It can be more efficient as it gets rid of oxygen dependence. With the surface photovoltage (SPV) technique introduced in the Supplementary Data, the generation of photogenerated holes on Nano-SA-TCPP under red light (500–800 nm) can be ascertained [41], as shown in Fig. 5a. It has been reported that the oxidation potential of the photogenerated holes in Nano-SA-TCPP is + 1.53 V [25], which is so strong that it can oxidize pollutant and water molecules. And the existence of photogenerated holes usually needs to depend on the semiconducting solid, and no single molecule can form photogenerated holes. Therefore, we also compared the performance of the Nano-SA-TCPP with the molecular PDT drug (Ce-6) and the molecular TCPP, as shown in Figs 5a and S21. Since these molecular drugs did not have a semiconductor band structure, no photogenerated hole signal could be detected under the same irradiation. Therefore, compared with Nano-SA-TCPP, molecular drugs have much weaker killing effect on cancer cells. It is also the biggest difference between photocatalytic cancer therapy by Nano-SA-TCPP and by the typical PDT.

The photogenerated-hole-induced cancer therapy by supramolecular Nano-SA-TCPP was further confirmed. The active species were captured by the method presented in the Supplementary Data, as shown in Fig. 5b. By comparing the killing effects after capturing different reaction intermediates’ inner cells, the main active species of the therapy can be determined. When the singlet oxygen was inhibited by NaN_3_, the killing effect did not change significantly, which indicated that singlet oxygen does not play a major role in the photocatalytic anticancer process. In other words, the photocatalytic therapy is not a traditional Type II PDT. However, when the photogenerated holes were inhibited by KI, the killing effect decreased significantly from 0.79 to 0.35 on average, which means that the photogenerated holes are the major intermediate in the novel therapy. And in the photocatalytic process, photogenerated holes interact with water or hydroxide to generate hydroxyl radicals (·OH), and photogenerated electrons interact with oxygen to generate superoxide radicals (·O_2_^−^) [42]. These free radicals also have oxidative killing effects on cells, which is similar to the Type I PDT. As shown in Figs 5c and S22, the ROS concentration increased in cells after irradiation. The increase of intracellular ROS could lead to apoptosis and destruction of genetic information [43]. When the (·OH) and (·O_2_^−^) were inhibited by 2-propanol and p-benzoquinone respectively, the killing effect was also decreased lightly. In short, the photogenerated holes conducted a powerful intracellular oxidation to kill cancer cells based on the nano photocatalysts inside.

Moreover, the photothermal effect was also excluded as the temperature of Nano-SA-TCPP dispersion after irradiation was far lower than the killing temperature of 42°C (Fig. S23), which proved the highly efficient therapy was not caused by the photothermal effect.

The reason for solid tumor elimination was further investigated. As shown in Fig. 5d the morphology of cancer cells after therapy changed significantly. The cell membrane was ruptured leading to the outflow of cytoplasm. As a result, the cells shrunk significantly and even broke into pieces. This result also matches the change in cell morphology and the decrease in the number of cells in the visual field shown in Fig. 2d. This conclusion has also been statistically verified with flow cytometry analysis. As shown in Fig. 5e and f, the cells before irradiation accumulated in the Q3 quadrant, which means that the cells were single body and dispersion in the buffer. However, the cell distribution, after photocatalytic therapy under 600 nm for 10 min, moved to Q4 quadrant, where the forward scatter (FSC) signal decreased obviously, indicating the decreased cell size. Besides, the side forward scatter (SSC) increased a little bit, indicating the cells might be broken into pieces [44].

From all the above, the photocatalytic therapy mechanism can be schemed as shown in Fig. 5g. The Nano-SA-TCPP can destroy the cell structure from the inside of cancer cells with the photogenerated holes under red light, causing the outflow of intracellular material, which is the reason for the flattened solid tumors after treatment as well. Due to this great advancement in the mechanism, Nano-SA-TCPP exhibits significant advantages in terms of therapeutic efficacy over conventional cancer phototherapy.

## CONCLUSION

In conclusion, a novel cancer therapy has been developed based on the supramolecular porphyrin photocatalyst. The solid tumors can be rapidly eliminated under red light. The therapy utilized the supramolecular photocatalysts to oxidize the cancer cells by photogenerated holes. The supramolecular photocatalyst can be enriched in cancer cells and tumors without causing damage to normal tissues and organs. Meanwhile, the supramolecular porphyrin photocatalyst can be completely metabolized by the organism without causing bioaccumulation. It can be expected that with the further enrichment of photocatalytic materials and advancements in mechanism understanding, photocatalytic cancer treatment will play an important role in conquering tumors.

## METHODS

### *In vitro* photocatalytic cancer therapy with Nano-SA-TCPP

Hela cells were also the probe cells to evaluate the cancer therapy performance of Nano-SA-TCPP. Specifically, Hela cells were incubated in the 25 cm^2^ cell-culture flask and then the cells (1 × 10^4^ cells per well) were seeded into two 96-well plates by detaching from the flask. After seeding, the Hela cells were exposed to Nano-SA-TCPP of different concentrations for 24 h.

After that, each well was irradiated under different wavelengths for 10 min, and during the irradiating other wells were kept in the dark with tinfoil. The light source was a xenon lamp source, PLS-SXE 300D, Beijing Perfectlight Technology Co., Ltd. with band-pass filters (600 ± 15, 650 ± 15, 700 ± 15 nm), and the irradiance was 0.1 W cm^−2^. For the cell viability after irradiation, a mixture of CCK-8 and DMEM (1 : 10) was added to the 96-plate. The cell viability was calculated as the ratio of the absorbance of the wells. The absorbance at 450 nm was measured by Thermo Multiskan FC. The cell flow cytometry analysis was conducted on a BD Biosciences FACSCalibur flow cytometer with the ROS probe of DCFH-DA purchased from Beyotime Biotechnology. The data were analyzed by FlowJo Software.

### *In vivo* photocatalytic cancer therapy with Nano-SA-TCPP

All animal procedure complied with the institutional animal regulations. The Male nude Balb/c mice were purchased from Beijing HFK Bioscience Co., Ltd. The 2 × 10^6^ Hela cells suspended in 200 μL DMEM were injected subcutaneously in the right lateral back of each mouse. The mice bearing Hela tumors were treated when the tumor volume was more than ∼100 mm^3^. All mice were randomized into three groups. The three groups of mice (n = 5) were dosed with 100 μL of phosphate buffer saline (PBS) and Nano-SA-TCPP (500 μg mL^−1^). The given amount of Nano-SA-TCPP was at a concentration per body weight of 2.50 mg kg^−1^ (3.16 μmol kg^−1^). Ten minutes later, the tumor site was irradiated under the light source with the 600 nm band-pass filter (0.1 W cm^−2^) for 10 minutes. After that, the tumor size and the body weight of the animals were monitored every day. No further light treatment was performed. The volume of the tumor was calculated by the equation of V = (L × W^2^)/2, where L is regarded as length and W is the width of the tumor measured using a caliper. After 16 days of therapy, the main organs and tumors were dissected and fixed in a 4% formaldehyde solution for 24 h at room temperature. The slices of the organs were stained with hematoxylin and eosin and investigated for histological variations.

## Supplementary Material

nwaa155_Supplemental_File
