# Supplementary material for: Photogenerated-hole-induced rapid elimination of solid tumors by the supramolecular porphyrin photocatalyst
Source: Natl Sci Rev. 2020 Jul 2;8(5):nwaa155. doi: 10.1093/nsr/nwaa155 (PMC8288340; doi:10.1093/nsr/nwaa155)
Supplement: nwaa155_Supplemental_File [file nwaa155_supplemental_file.pdf]

Supplementary data for

Photogenerated holes induced rapid eliminating of solid tumors by the supramolecular porphyrin photocatalyst

Zijian Zhang[1], Li Wang[2], Weixu Liu[1], Zihe Yan[1], Yongfa Zhu[1]*, Shuyun Zhou[2] and Shanyue Guan[2]*

Correspondence to: zhuyf@mail.tsinghua.edu.cn,

guanshanyue@mail.ipc.ac.cn

This PDF file includes:

Methods

Figures S1 to S23

Captions for Movies S1

Methods

Synthesis of Nano-SA-TCPP

Tetra(4-carboxyphenyl)porphyrin (TCPP) was purchased from TCI Shanghai. 0.700 g of TCCP was dissolved with 25 mL 1 mol L^{-1} KOH aqueous solution in a 500mL flask. The solution was heated until the solid was fully dissolved, which can be observed by light exposure. 0.1 mol L^{-1} HCl was added dropwise by a constant pressure dropping funnel until the pH changed into neutral and no further solid was precipitated. And the mixture was allowed to stand and cooled to room temperature. After cooling down, the mixture needs to be centrifuged and washed with water several times to remove potassium and chloride ions. Then, the clean Nano-SA-TCCP was collected by filtration and dried with a vacuum under 60 °C.

Characterizations

The ultrasonic image was observed on High Resolution Imaging System, Vevo770, in Core Facility Center of Capital Medical University, Beijing. The fluorescence imaging was observed by Lumina III, PerkinElmer. The surface photovoltage was measured by a made-up instrument as previously reported [1]. The high-resolution transmission electron microscopy (HRTEM) images were obtained by a JEM 2010F field emission gun transmission electron microscope with an accelerating voltage of 200 kV. Fourier transform infrared (FTIR) spectra were carried out using Bruker V70 FTIR spectrometer. The contact angle was conducted on the OCA15 pro, Dataphysics, Germany. The

Zeta potential and particle size were measured with nanoparticle analyzer SZ-100, Horiba.

Measurement of Surface Photovoltage

The monochromatic light resource was a 500 W xenon lamp, CHF XQ500W, Global xenon lamp power, with a double-prism monochromator, Omni-λ 3005. The slit width was set at 3 mm. The photovoltage signal was amplified by a lock-in amplifier, SR830-DSP with a light chopper, SR540. The resolution of the spectrum was 1 nm. The raw SPV data were normalized using the illuminometer, Zolix UOM-1S.

***In vitro* cytotoxicity of Nano-SA-TCPP**

In vitro cytotoxicity of Nano-SA-TCPP was tested on the human Hela cells. Specifically, Hela cells were incubated in the 25 cm^2 cell-culture flask and then the cells (1×10^4 cells per well) were seeded into two 96-well plates by detaching from the flask. After seeding to the 96-plates, we incubated the Hela cells to a series dose of Nano-SA-TCPP for 24 h. We further mixed the CCK-8 and DMEM (1:10) and then added to the 96-plates with further 24 h incubation. The cell viability was calculated as the ratio of the absorbance of the wells. The absorbance at 450 nm was measured by Thermo Multiskan FC.

In vitro fluorescence imaging of Nano-SA-TCPP

To investigate the *in vitro* imaging performance of Nano-SA-TCPP, 1×10^6 Hela cells were seeded into a plate for 24 h at 37 °C. Then, 25 μg mL^{-1} of Nano-SA-TCPP sample was added into the plate with further incubated for 24 h. Then the cells were washed with PBS three times and its fluorescence imaging documented by Nikon A1R Eclipse Ti confocal laser scanning microscope with a 40× water-immersion objective.

Active species capture *in vitro*

Hela cells were also the probe cells to evaluate the cancer therapy performance of Nano-SA-TCPP. Specifically, Hela cells were incubated in the 25 cm^2 cell-culture flask and then the cells (1×10^4 cells per well) were seeded into two a 96-well plate by detaching from the flask. After seeding 1×10^4 cells/well to a 96-plate, we exposed the Hela cells to a series of capturers (1mg mL^{-1}, 100μL) and 25 μg mL^{-1} of Nano-SA-TCPP for 24 h.

For the specific capturers, sodium azide (NaN_3) was used as singlet oxygen ($^1O_2$), isopropanol was used as hydroxyl radicals (·OH) capturer, p-benzoquinone was used as superoxide radicals ($\cdot O_2^-$) capturer, and potassium iodide (KI) was used as photogenerated holes (h^+) capturer.

After that, each well was irradiated under different wavelength for 10 min, during the irradiating, other wells were kept in dark with tinfoil. The light source was the same as ***In vitro* Photocatalytic cancer therapy**. For the cell

viability after irradiation, the mixture of CCK-8 and DMEM (1:10) was added to the 96-plate. To exclude the toxicity of the capturers, the control groups (incubated with capturers without irradiation) were also set up. The cell viability was calculated as the ratio of the absorbance of the wells. The absorbance at 450 nm was measured by Thermo Multiskan FC.

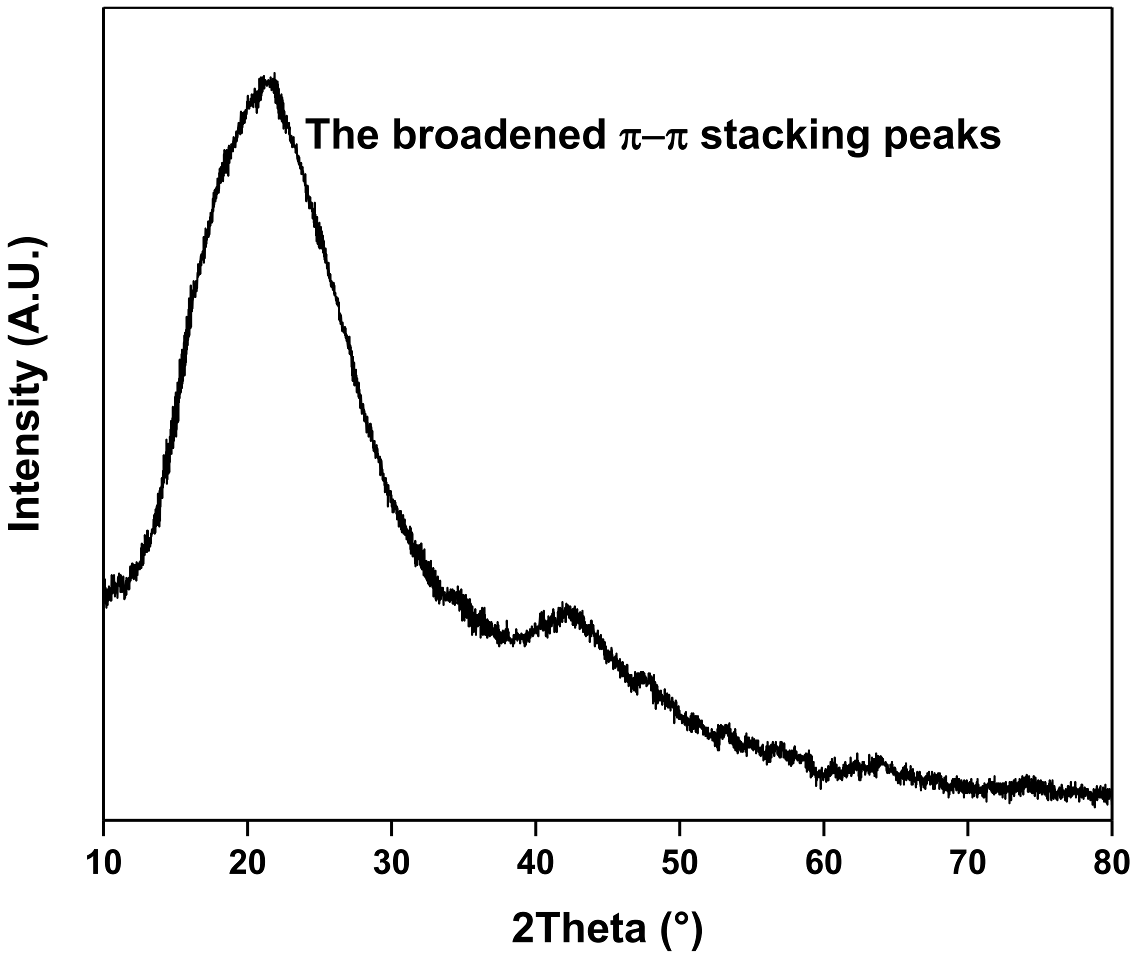

Figure S1. The X-ray diffraction spectrum of the Nano-SA-TCPP. The XRD diffraction peaks of supramolecular materials are completely consistent with our previous reports [2]. The broadened peak in the range of 20-30° can be attributed to the characteristic π-π stacking inner Nano-SA-TCPP. At the same time, the broadening of the peak is due to the nanocrystal size.

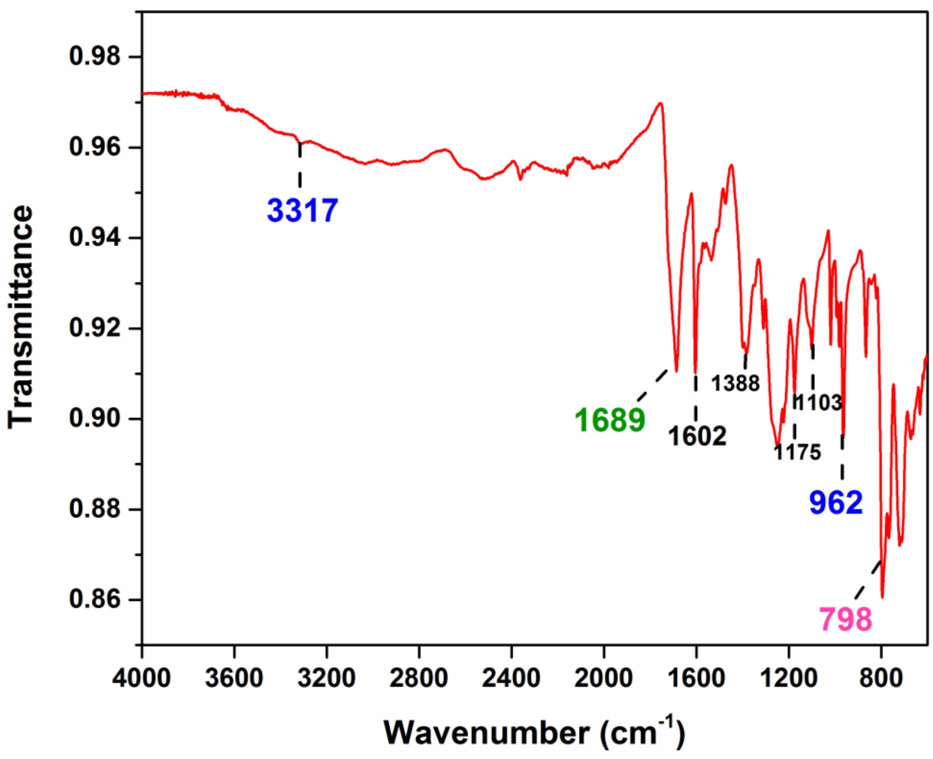

Figure S2. IR spectrum of the Nano-SA-TCPP. The peaks can match with the result we previously reported [2] very well, which indicated the correction of our synthesis. FT-IR (ATR):3317, 1689, 1602, 962, 798 cm^{-1}.

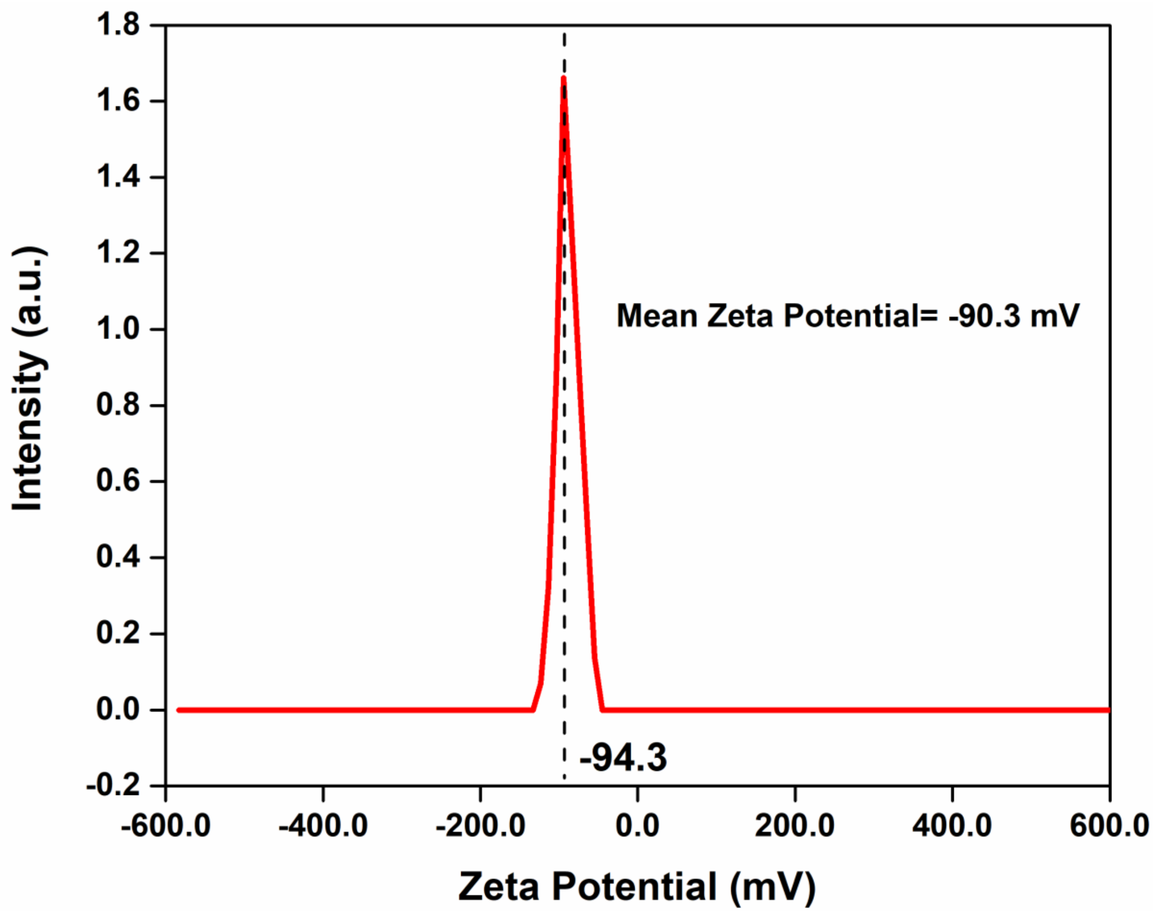

Figure S3. Zeta Potential of Nano-SA-TCPP. The max intensity appeared at the potential of -94.3 mV. And after statistic calculation, the mean zeta potential of Nano-SA-TCPP was -90.3 mV. The negative potential is helpful for the internalization behaviors of Nano-SA-TCPP [3].

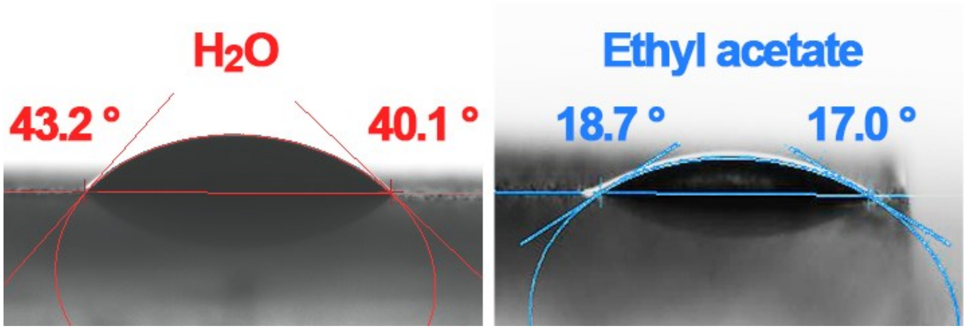

Figure S4. Contact Angles of H_2O and Ethyl Acetate on Nano-SA-TCPP. The Nano-SA-TCPP presents good amphipathicity. Firstly, the Nano-SA-TCPP dispersion was dip-coated on a substrate of glass to form a thin film. After that, a drop of water and ethyl acetate were dropped on the film. It can be observed the Nano-SA-TCPP presents out-standing hydrophilic property, where water can infiltrate the film, resulting in small contact angles of 43.2° and 40.1°. At the same time, the contact angles between ethyl acetate and Nano-SA-TCPP are much smaller of 18.7° and 17.0°, which proved the lipophilicity. It is important that the amphipathic property of Nano-SA-TCPP contributed to the uniform aqueous dispersion that can be injected into the experimental mice. And the lipophilicity is also benefited for the Nano-SA-TCPP to be taken into cells.

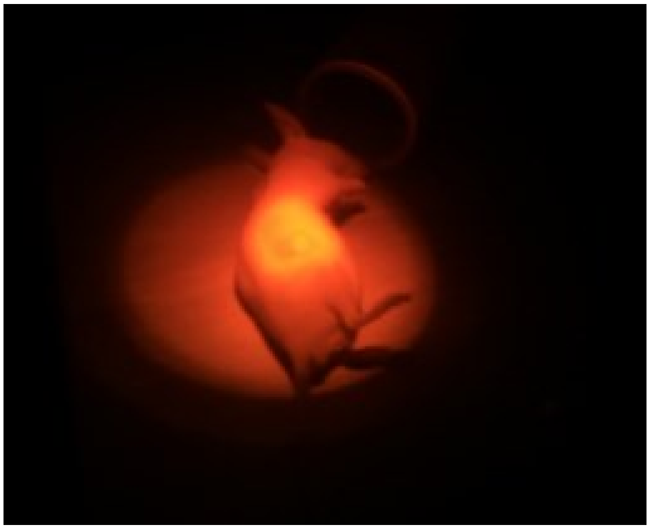

Figure S5. The photograph of Photocatalytic cancer therapy processes in vivo. The mouse was anesthetized by gas. After that, it was irradiated for 10 min with 600 nm red light. The power of light on the skin was 0.1 W cm $^{-2}$.

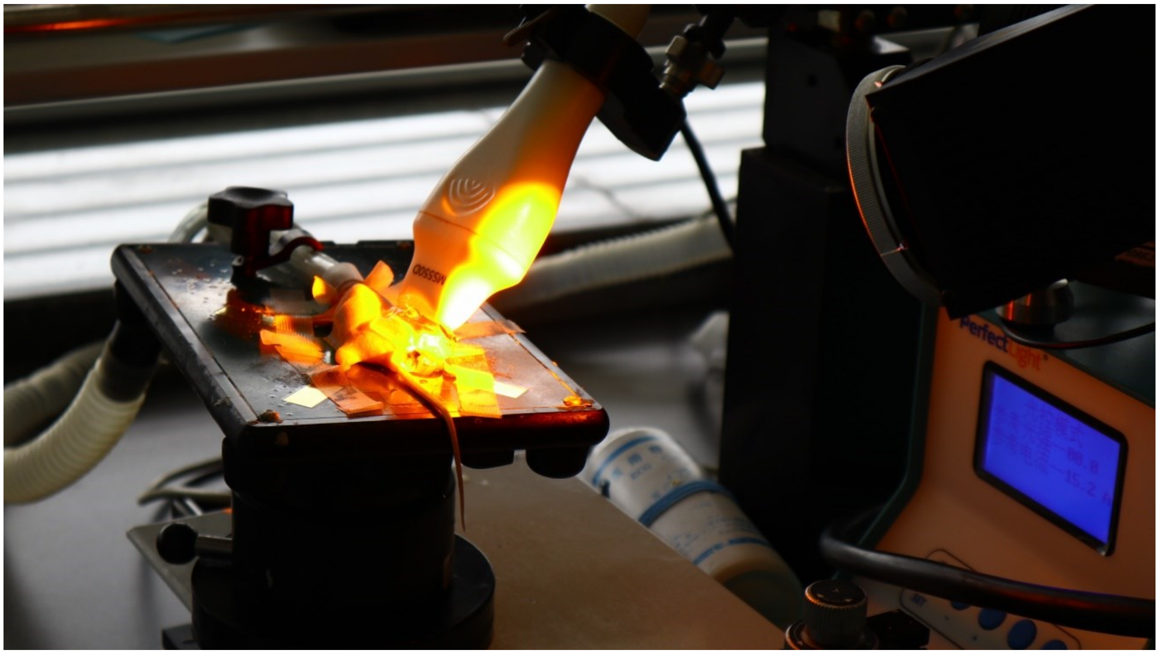

Figure S6. Photograph of in-situ ultrasonic imaging to the photocatalytic therapy in vivo. The light source is the xenon lamp source with a band-pass filter of 600 nm, PLS-SXE 300D, Beijing Perfectlight Technology Co., Ltd.

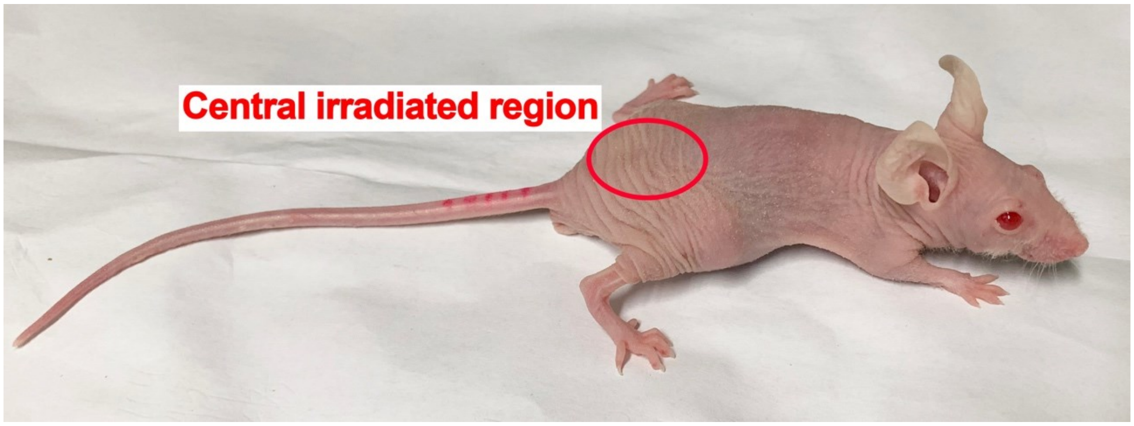

Figure S7. Photograph of a healthy mouse after irradiation. The light source is the xenon lamp source with a band-pass filter of 600 nm, PLS-SXE 300D, Beijing Perfectlight Technology Co., Ltd. There was no obvious change on the skin of the mouse, which indicated that the tumor elimination effect was not contributed by the light irradiation alone. And the result also proved the safety of the light source.

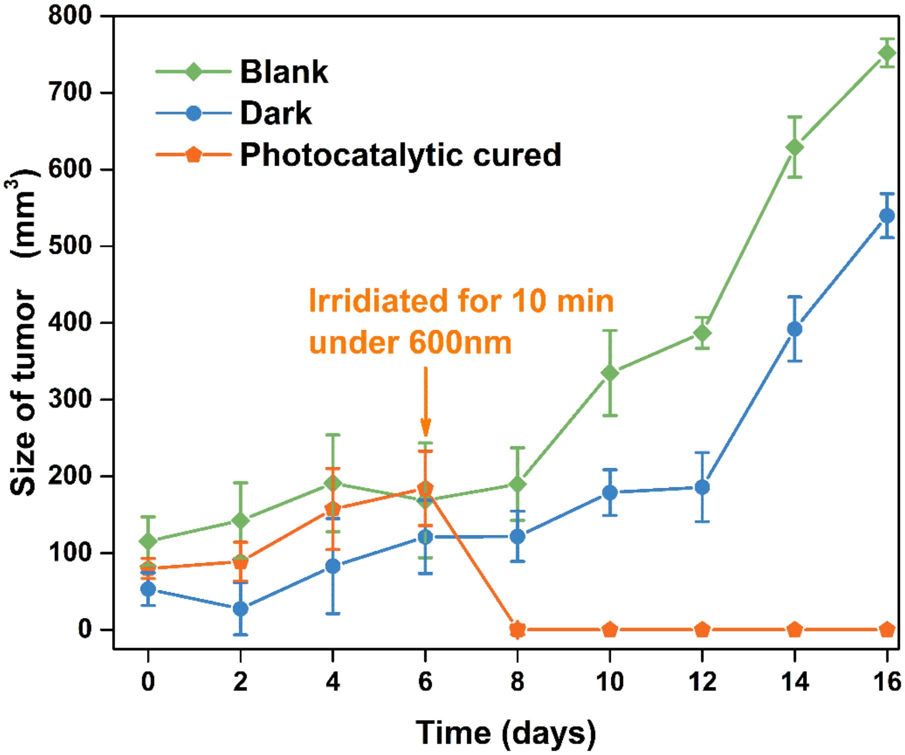

Figure S8. The absolute size of the tumor in vivo with&without therapy. The photocatalytic cancer therapy was conducted after the tumor size exceeded 100 mm^3. After treatment, the tumor was cleared completely, while the tumor size of two control groups increased crazily. Especially for the Dark group, which was also injected with the same amount of Nano-SA-TCPP dispersion but protected from light, the terrible prolife of tumor also proved the biosafety of Nano-SA-TCPP and the switch to the toxicity of light.

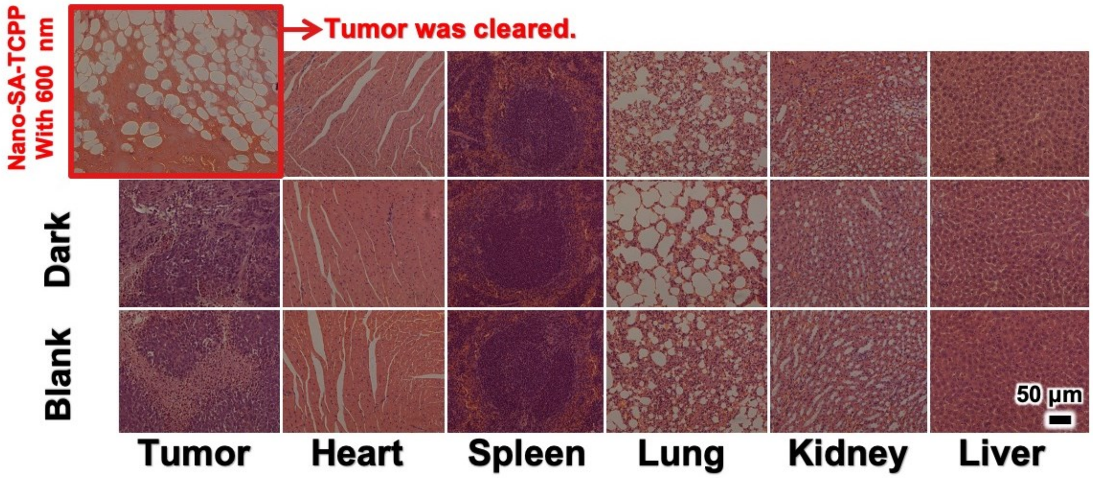

Figure S9. The sectioning results of tumors and main organs. Tissue sections also confirmed that biocompatible Nano-SA-TCPP did not harm the main organs after entering the body, but only to kill cancer cells under the excitation of red light.

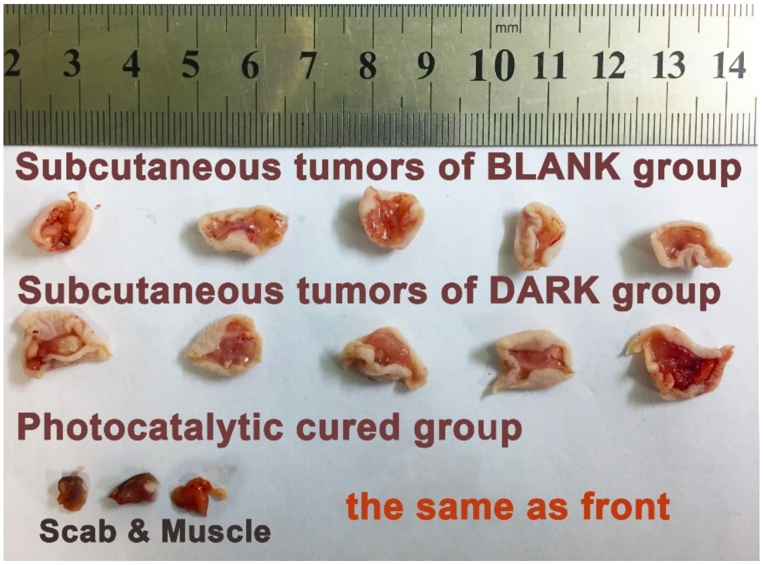

Figure S10. Photograph of tumors with/without photocatalytic treatment, the Blank group is consisted of the mice without Nano-SA-TCPP, and the Dark group consists of the mice without irradiation, which was injected of Nano-SA-TCPP in the same amount as the experimental group. In the control groups, subcutaneous tumors were observed, while in the photocatalytic cured group, the tumor has been cleared completely, only scab and muscle left.

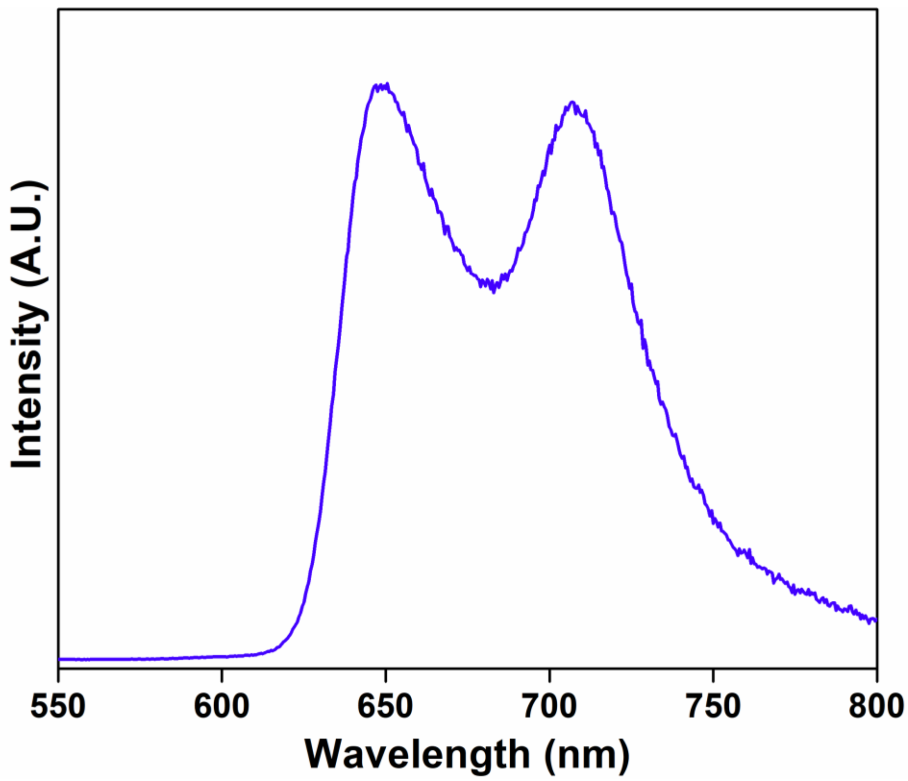

Figure S11. Fluorescence emission spectroscopy of Nano-SA-TCPP dispersion. There are two obvious peaks at 648 and 706 nm, which are excited by 408 nm. The long wavelength of emission fluorescence provides a tool to monitor Nano-SA-TCPP *in vivo*. And it also inspired us a property of integrated performance of diagnosis and treatment via Nano-SA-TCPP. With fluorescence imaging, the targeted tumor site can be selected and irradiated directly.

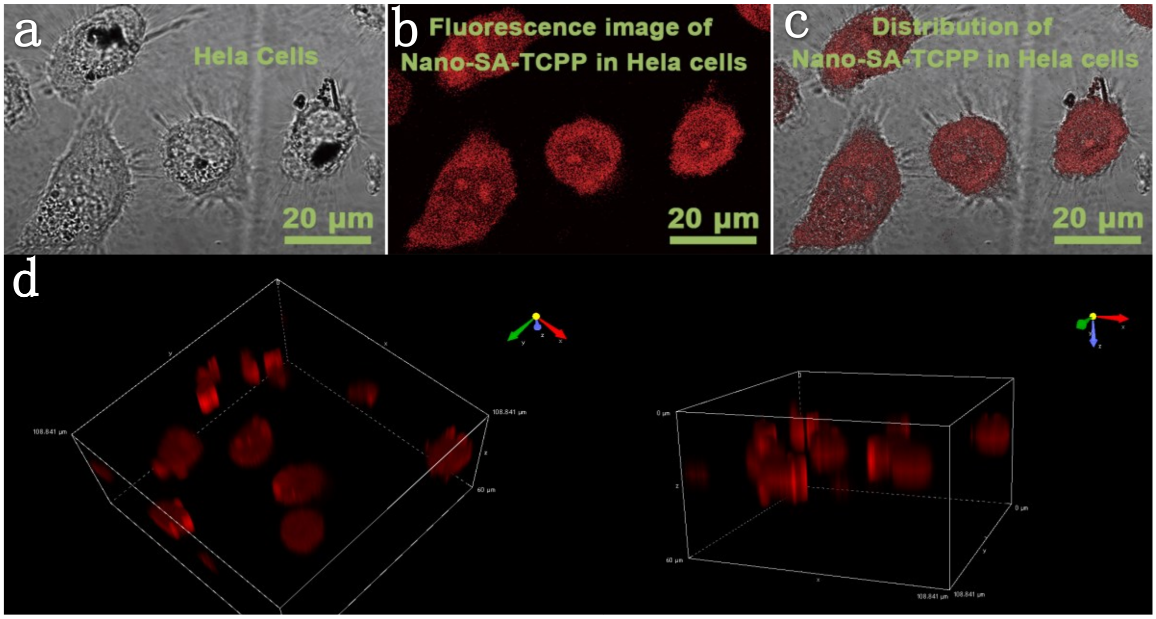

Figure S12. The fluorescence images of Nano-SA-TCPP in Hela cells. (a) The bright-field image of Hela cells; (b) fluorescence images of Nano-SA-TCPP in Hela cells collected by 703 nm fluorescence of Nano-SA-TCPP; (c) Overlap of (a) and (b), which indicated the Nano-SA-TCPP was uptaken into cells. (d)The 3D confocal images of Hela cell incubated with 25μg mL^{-1} of Nano-SA-TCPP for 24 h. The fluorescence signal can be detected in the 3D scale of cells, which proves that the Nano-SA-TCPP get into cells and distribute evenly, not just adsorbed on the cell membrane.

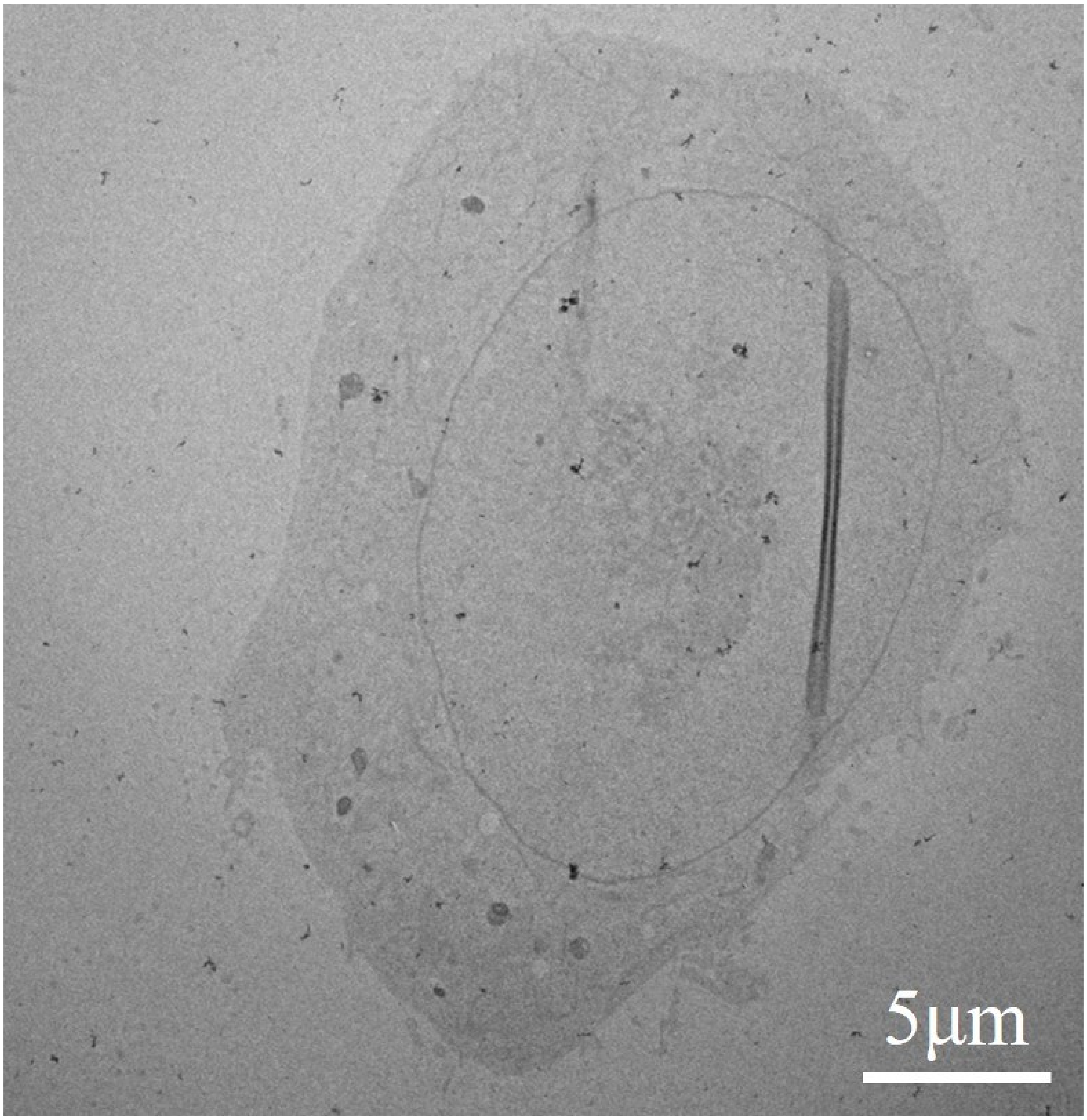

Figure S13. TEM image of the whole Hela cell in cellular uptake processes. It can be observed that the morphology of the cell after incubation with Nano-SA-TCPP maintains well completeness, which means the Nano-SA-TCPP did not lead to the toxicity.

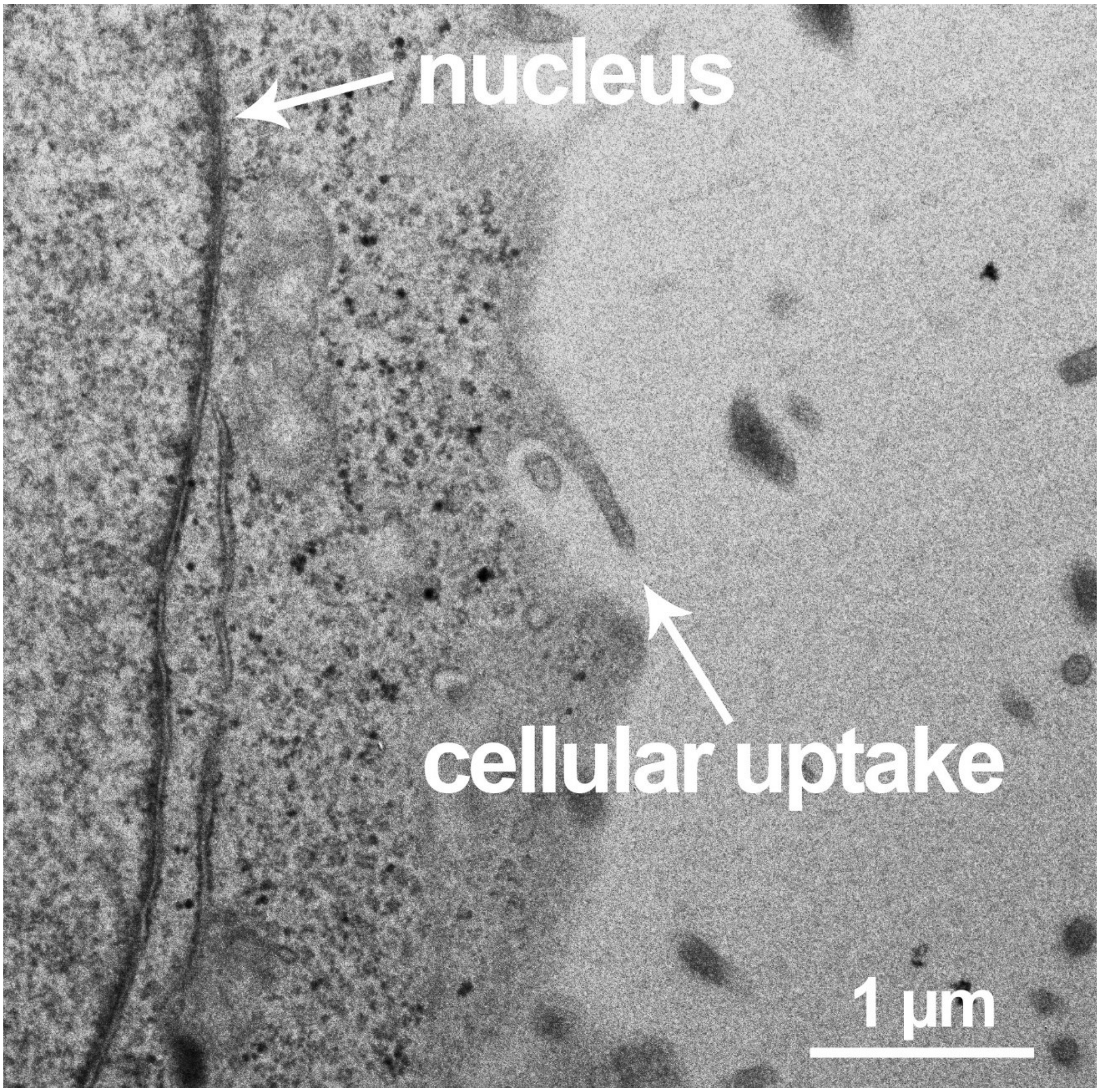

Figure S14. TEM image of Hela cell in cellular uptake processes. It can be seen that the cell membrane formed a depressed structure for cellular uptake of Nano-SA-TCPP.

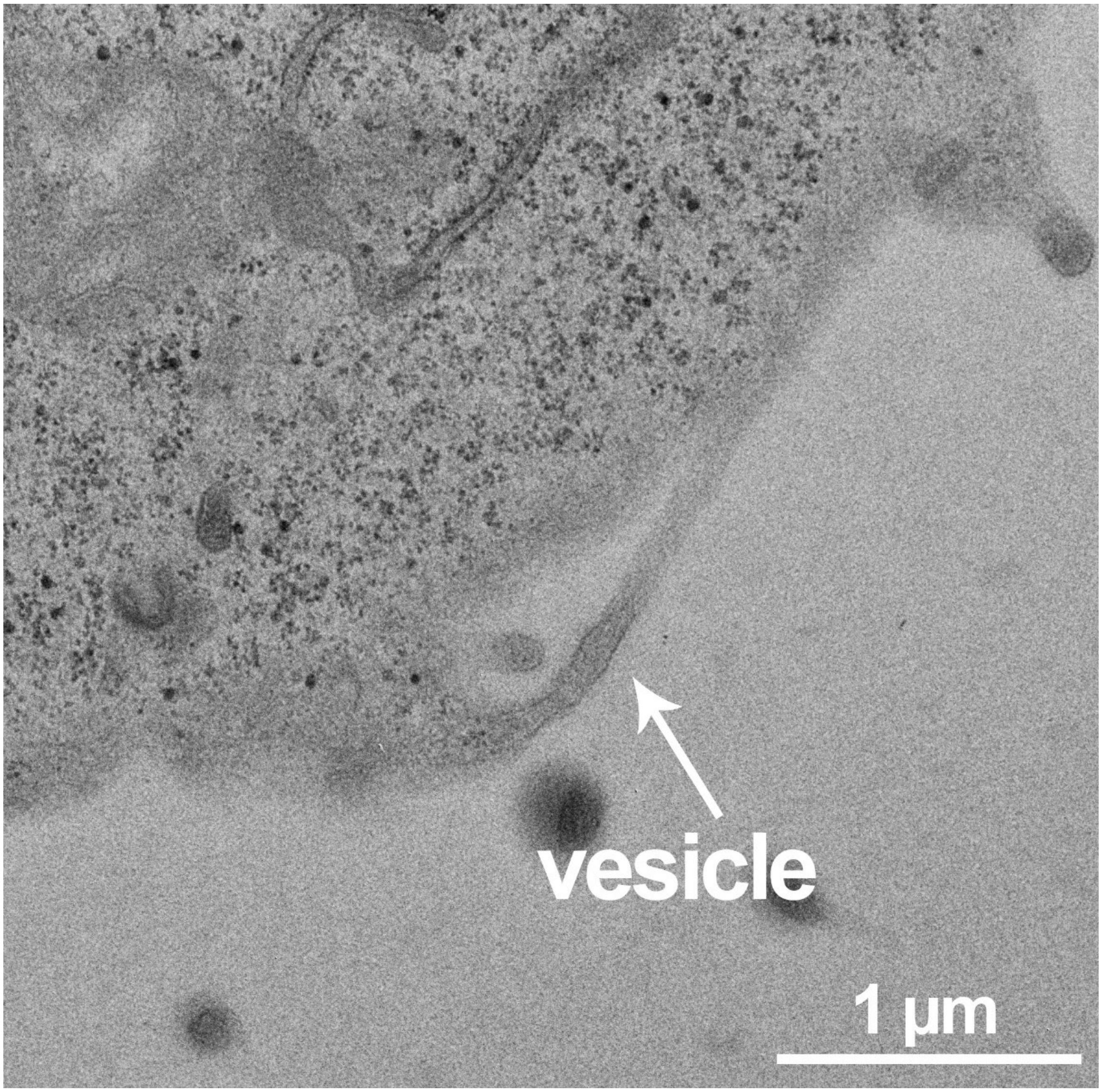

Figure S15. TEM image of Hela cell with internalized Nano-SA-TCPP. The cell structure is obvious. It can be seen that the Nano-SA-TCPP was surrounded in a vesicle.

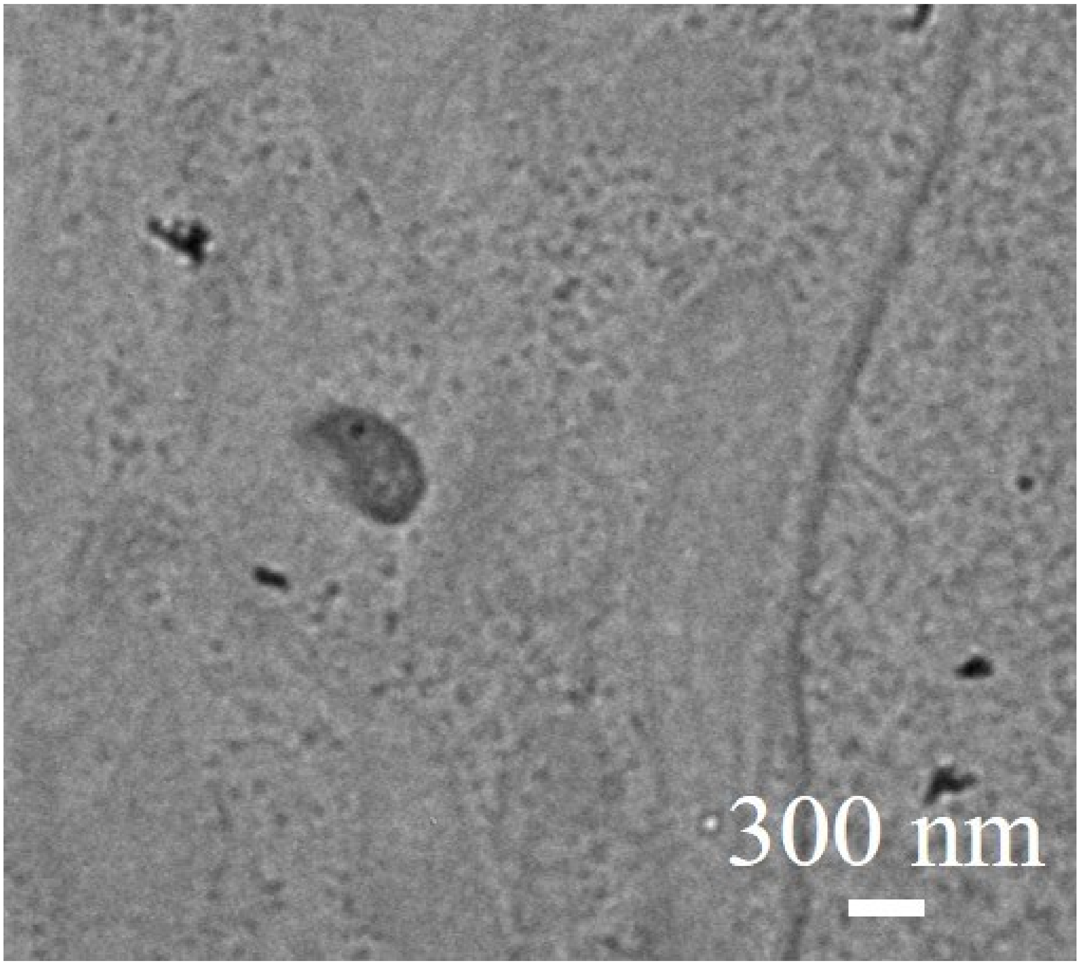

Figure S16. TEM image of Hela cell with internalized Nano-SA-TCPP with a closer look. According to the previous paper reported [4,5], the nanomaterials can occur slightly gather in the cell via endocytosis and thus particles looked bigger than 50 nm in the cell. It can be found that the particle size of the material is about 300 nm, indicating that the particles occur slightly gather into the cell.

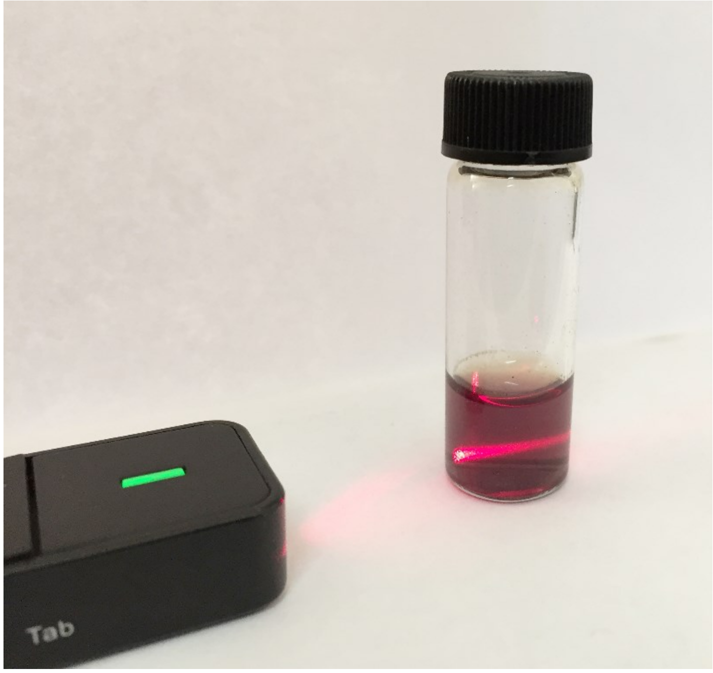

Figure S17. The photograph of 500 μg mL^{-1} Nano-SA-TCPP dispersion liquid. With laser irradiation, the Tyndall effect can be observed clearly, which indicates that Nano-SA-TCPP is not dissolved in water to form a homogeneous solution. In other words, Nano-SA-TCPP was injected into the tumor in the form of nanocrystal photocatalysts, which is the foundation of photogenerated holes. Photogenerated holes must depend on the semiconductor solids, which cannot be transported through a solution or other medium.

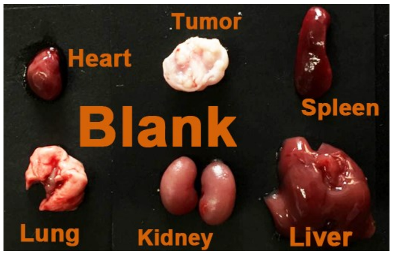

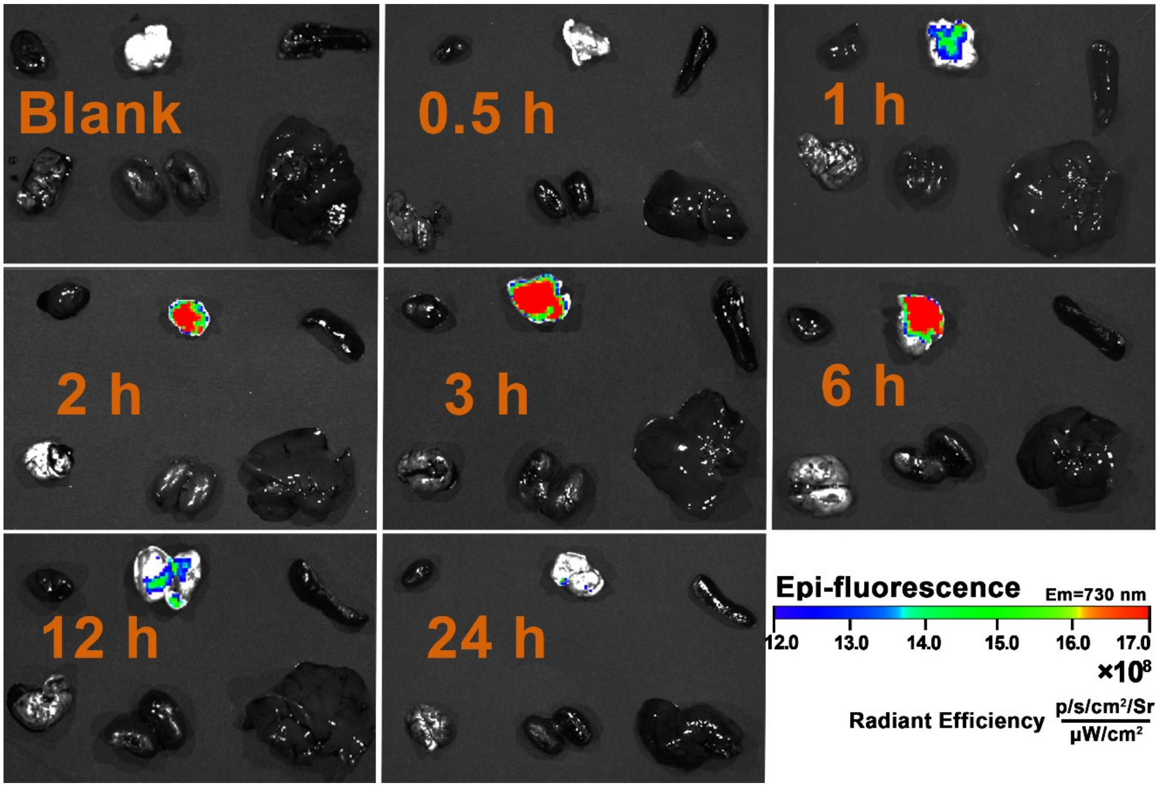

Figure S18. The time-dependent Nano-SA-TCPP distribution in the main organs and tumor site observed by fluorescence images. We can clearly observe the process of accumulation and metabolization of materials in the tumor site. After injection, the drug was gradually enriched in the tumor site and reached its maximum in 6 hours. Subsequently, the drug began to be metabolized and completely metabolized after 24 hours.

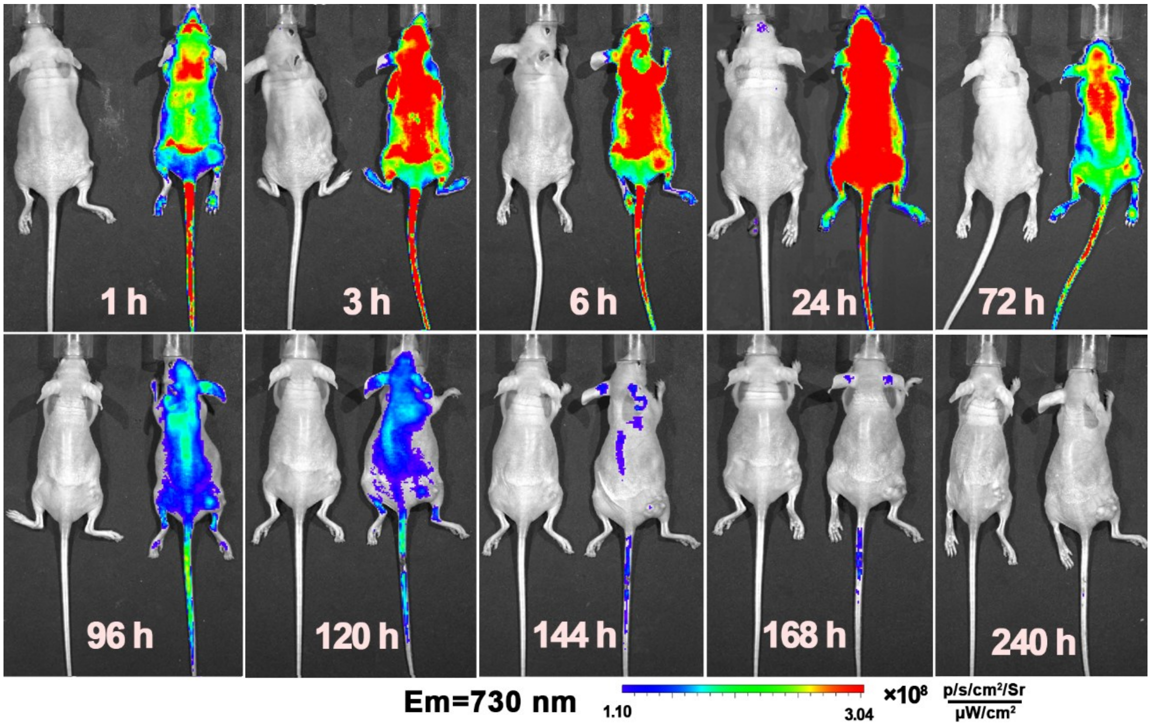

Figure S19. Fluorescence imaging of Nano-SA-TCPP *in vivo* with the 730 nm emission peak. The time-dependent SA-TCPP distribution *in vivo* observed by fluorescence image, where 100 μL of Nano-SA-TCPP dispersion (500 μg mL^{-1}) was injected into mice through the tail vein. The equivalent injection volume is 2.50mg kg^{-1} BW (3.16μmol kg^{-1} BW). It can be observed that the Nano-SA-TCPP reached the highest concentration in the whole body one day after injection. After that, the SA-TCPP was accumulated in the tumor site, proved the targeted properties. Finally, it can be degraded within 10 days, which means the Nano-SA-TCPP did not lead to the accumulative toxicity.

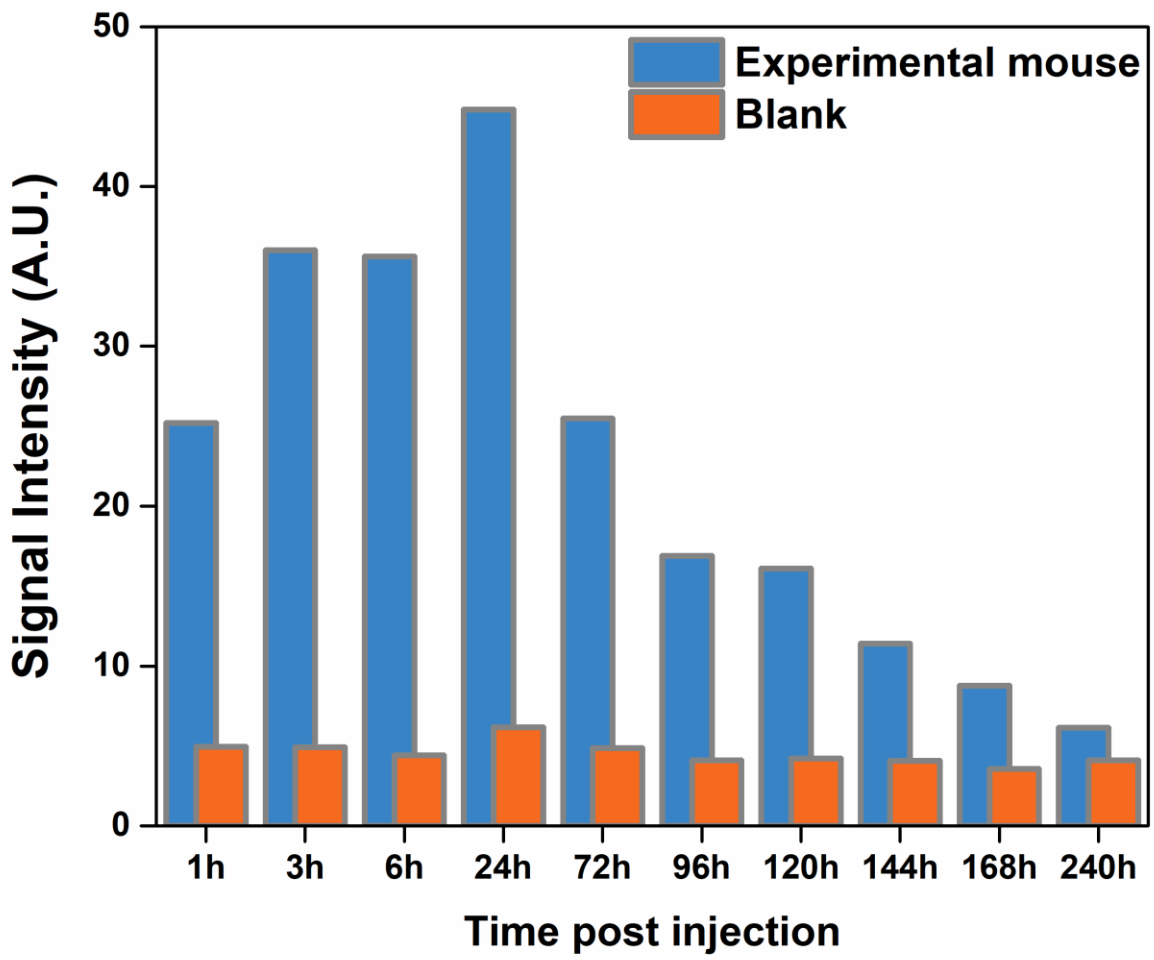

Figure S20. Fluorescence intensity statistics of Figure S19. It can be calculated that at the 24 h after injection, the fluorescence intensity reached the maximum (44.8) which was 9.85 times higher than the average background intensity (4.55).

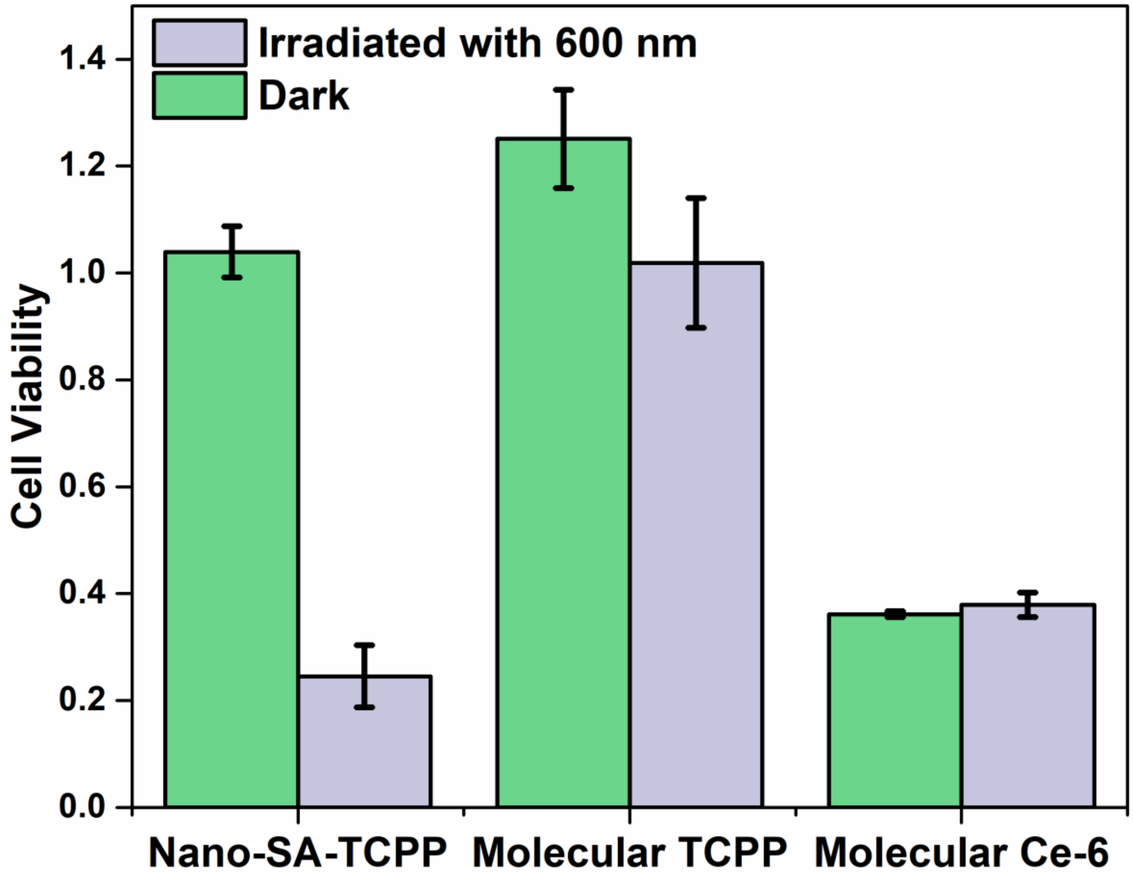

Figure S21. Comparison of Nano-SA-TCPP and molecular drugs for cancer therapy *in vitro*. Nano-SA-TCPP and molecular TCPP powder were 25 μg mL^{-1} in dispersion, and the concentration of Ce-6 was 18.9 μg mL^{-1}, same to Nano-SA-TCPP in mole. The molecular TCPP cannot produce photogenerated holes thus which cannot kill the cancer cells efficiently. While, to the clinical used Ce-6 porphyrin, it presents obvious cellular toxicity in the same amount.

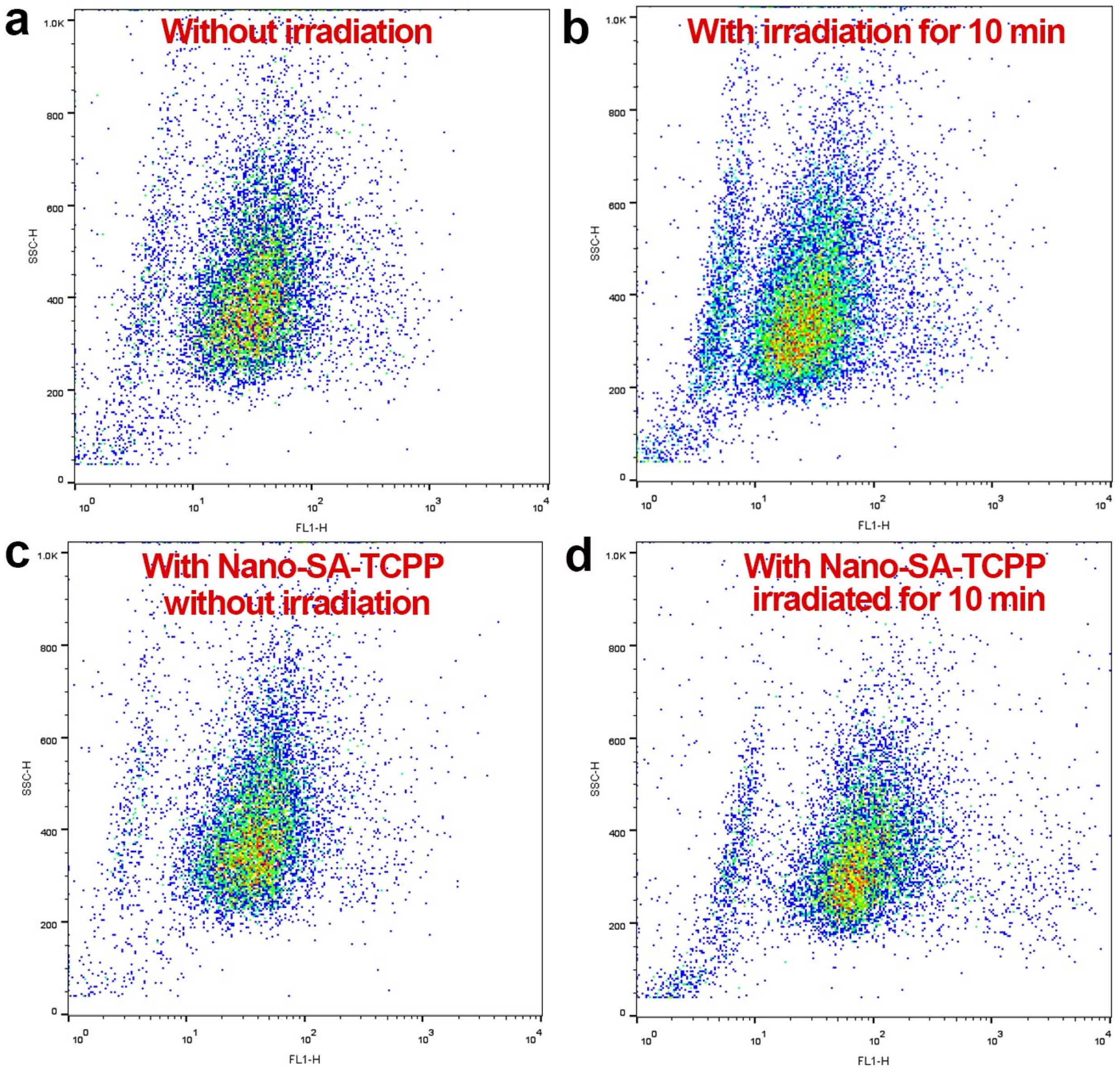

Figure S22. Dot plot of SSC in the ROS detection in vitro. (a) The dot plot of Blank cells without irradiation; (b) The dot plot of Blank cells irradiated for 10 min; (c) The cells incubated with the Nano-SA-TCPP without irradiation; (d) The cells incubate with the Nano-SA-TCPP irradiated for 10 min.

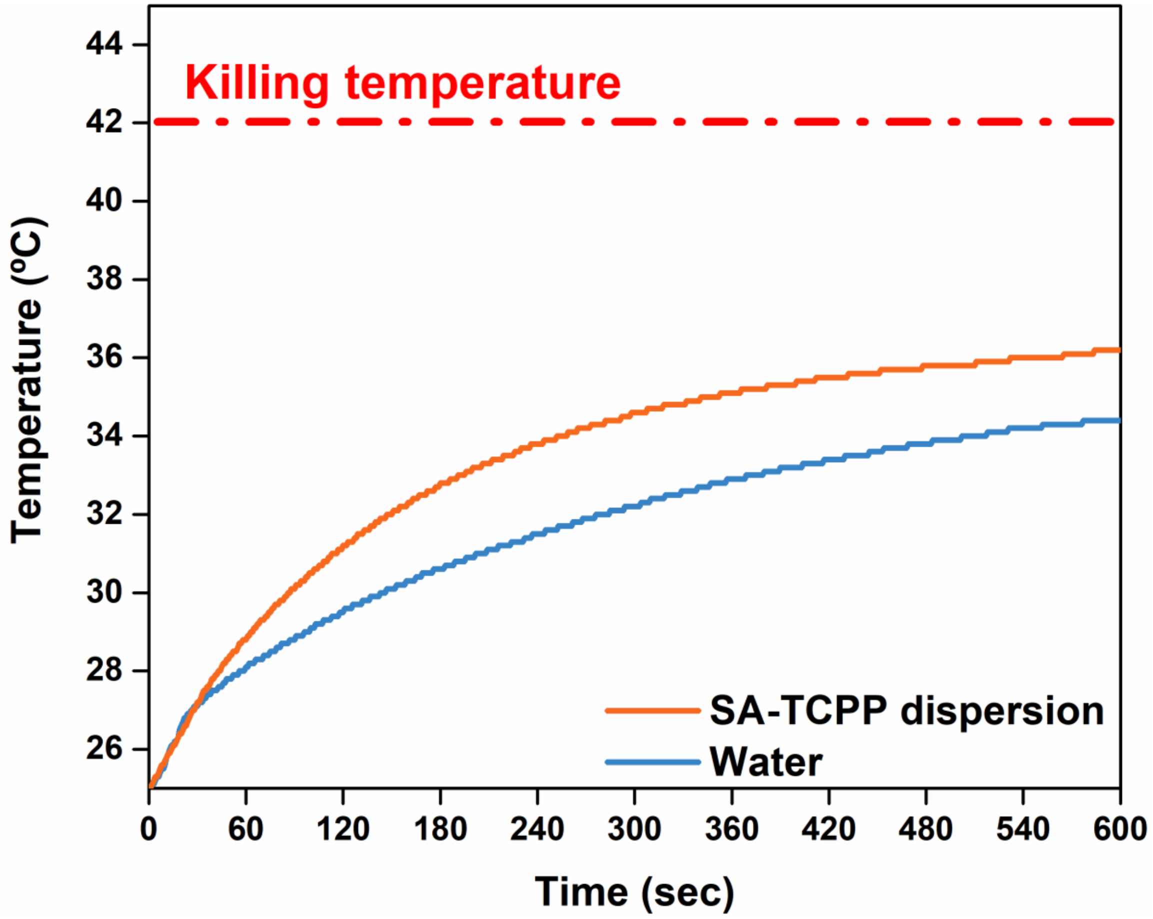

Figure S23. The photothermal effect of photocatalytic therapy. As shown with the curve, the Nano-SA-TCPP did not present an obvious photothermal effect, whose temperature after irradiation was far below the cell-killing temperature (42 °C).

Capitation for Movie S1

The Ultrasonic in-situ monitoring during photocatalytic therapy. It can be seen that at the beginning of the therapy, there is an obvious strong ultrasonic echo signal, indicating a solid tumor tissue. As the irradiation carried on, the echo signal intensity decreased rapidly. After 10 minutes, the origin solid tumor echo signal cannot be detected, at the same time, the convex site was flattened. Thus, the solid tumor was eliminated within 10 min.

References

1. Li S, Meng D and Hou L *et al*. The surface engineering of CdS nanocrystal for photocatalytic reaction: a strategy of modulating the trapping states and radicals generation towards RhB degradation. *Appl Surf Sci* 2016; **371**:164.

2. Zhang Z, Zhu Y and Chen X *et al*. A full-spectrum metal-free porphyrin upramolecular photocatalyst for dual functions of highly efficient hydrogen and oxygen evolution. *Adv Mater* 2019; **31**: 1806626.

3. Zhao F, Zhao Y and Liu Y *et al*. Cellular uptake, intracellular trafficking, and cytotoxicity of nanomaterials. *Small* 2011; **7**: 1322-1337.

4. Wang L, Sun Q and Wang X *et al*. Using hollow carbon nanospheres as a light-induced free radical generator to overcome chemotherapy resistance. *J Am Chem Soc* 2015; **137**: 1947-1955;

5.Wang L, Zhang T and Li P *et al*. Use of synchrotron radiation-analytical techniques to reveal chemical origin of silver-nanoparticle cytotoxicity. *ACS Nano* 2015; ***9***: 6532-6547.
